# Modern contraceptive use in Somalia: a multivariable analysis of prevalence and predictors among women of reproductive age

**DOI:** 10.3389/fgwh.2025.1689712

**Published:** 2026-01-12

**Authors:** Fatima Mohamud Ahmed, Abdifetah Ibrahim Omar, Hassan Abdi Ahmed

**Affiliations:** 1 Center for Graduate Studies, Department of Statistics and Data Analytics, Jamhuriya University of Science & Technology (JUST), Mogadishu, Somalia; 2 Faculty of Medicine and Health Sciences, Jamhuriya University of Science and Technology, Mogadishu, Somalia

**Keywords:** awareness and health education, modern contraceptive use, reproductive health, Somalia, uptake

## Abstract

**Background:**

Despite global advancements in reproductive healthcare, the utilization of modern contraceptives in Somalia remains critically low, marked by significant regional and sociodemographic disparities. This study assessed the prevalence and identified key predictors of modern contraceptive use among Somali women of reproductive age using data from the 2020 Somali Health and Demographic Survey (SHDS).

**Methods:**

A nationally representative cross-sectional analysis was conducted on a sample of 2,704 women aged 15–49 years. Descriptive statistics and multivariable logistic regression were employed to identify factors associated with the use of modern contraceptives.

**Results:**

The majority of participants were aged 26 years or older (57.6%), resided in urban areas (85.1%), and belonged to the highest wealth quintile (62.4%). The prevalence of modern contraceptive use among the study participants was exceptionally low at 1.8%. The most commonly reported methods were oral contraceptive pills (0.6%) and implants (0.2%), while the use of intrauterine devices (IUDs) was minimal (0.04%). A significant gap in exposure to family planning information was observed, with only 13.6% of women reported to have received it at health facilities. Key predictors for modern contraceptive use included the age of the women and exposure to family planning education at a health facility. Women aged 26 years and older demonstrated significantly higher odds of using contraception compared to their younger counterparts [adjusted odds ratio (AOR): 10.13; 95% confidence interval (CI): 2.39–42.97]. Furthermore, women who received family planning information from health facilities were twice as likely to use modern contraceptive methods (AOR: 1.99; 95% CI: 1.02–3.88).

**Conclusion:**

The findings underscore an urgent need to enhance both the accessibility and knowledge of modern contraceptives in Somalia. Targeted interventions focusing on health facility-based education and expanding the limited variety of available contraceptive methods are crucial to improving uptake and addressing the reproductive health needs of Somali women.

## Introduction

Modern contraceptive methods represent essential products in reproductive healthcare, enabling individuals to prevent unintended pregnancies and make informed decisions about when and whether to have children (1). Moreover, some modern contraceptives—such as male and female condoms—offer the dual benefit of preventing both pregnancy and the transmission of sexually transmitted infections (STIs) (2). These methods are generally categorized as hormonal (e.g., pills, injectables, implants), barrier (e.g., condoms), natural (e.g., fertility awareness), and permanent (e.g., sterilization) (3).

Despite global efforts and the availability of various contraceptive options, access to modern contraception remains limited, especially in low-resource settings. As of 2023, approximately 1.9 billion women aged 15–49 years required family planning services, yet only 842 million were using contraceptives. Alarmingly, an estimated 257 million women still lack access to these essential services (4, 5). Although global contraceptive coverage rose modestly from 76.5% to 77.6% between 2015 and 2023, Sub-Saharan Africa continues to lag behind, with only 57.4% of women having their family planning needs met (5, 6). This region accounts for nearly 70% of global maternal deaths, with a maternal mortality ratio of 454 per 100,000 live births as of 2023 (6, 7). Several factors contribute to this gap, including poor service availability, inadequate contraceptive choice, fear of side effects, sociocultural and religious resistance, and gender inequalities (5, 7). These challenges are widespread in Sub-Saharan Africa, where low contraceptive use continues to drive high fertility rates, as well as increased maternal morbidity and mortality (7, 8). Expanding access to modern contraceptive use is not only a public health priority but also a key strategy for reducing these maternal deaths, improving maternal and child health outcomes, reducing poverty, and promoting gender equality, all of which are critical to achieving the Sustainable Development Goals (SDGs) (2, 9–12).

In Somalia, modern contraceptive use is among the lowest globally (13). According to the 2020 Somali Health and Demographic Survey (SHDS), only 1.6% of women aged 15–49 years reported using contraceptives, while 6.9% expressed future intention and a staggering 91.5% had no intention of using any contraceptive method (14). The country's unmet need for contraception stands at 37%, well above global averages (15). This limited uptake has been associated with high fertility rates and poor birth spacing, contributing significantly to Somalia's maternal mortality ratio of 692 deaths per 100,000 live births, among the highest in the world (16, 17).

Despite global commitments to reproductive health, the use of modern contraceptives in Somalia remains critically low, contributing to persistently high maternal and child mortality. To explore the factors driving this low uptake and identify key sociodemographic determinants of modern contraceptive use, we analyzed nationally representative data from the 2020 SHDS (14, 15). Our findings provide an evidence-based roadmap for expanding access to family planning services and informing policies aimed at improving reproductive health equity across Somalia.

## Methods

### Study area

This study was conducted in Somalia, a country in the Horn of Africa, characterized by persistent conflict, fragile health infrastructure, and sociocultural norms that heavily influence reproductive behavior (18). These challenges, combined with widespread poverty and limited access to formal education and healthcare services, have created a complex environment for reproductive health interventions. The SHDS 2020 provides nationally representative data on these issues, making it an essential source for analyzing modern contraceptive use in Somalia (14, 17).

### Data source and study design

The analysis utilized data from the 2020 SHDS, conducted by the Somali National Bureau of Statistics (14). The SHDS was designed as a nationally representative cross-sectional survey. It collected data from 18 regions, stratified into urban, rural, and nomadic areas, with the exception of Banadir, which was considered entirely urban. Due to security concerns, three strata of Lower Shabelle and Middle Juba, as well as the rural and nomadic strata of Bay, were excluded (17). After cleaning the data in Stata, a total of 2,704 data points were analyzed in SPSS version 24.

### Variables

The dependent variable was current use of modern contraceptive methods, measured as a binary outcome (1 = user; 0 = non-user). Modern methods were defined according to WHO guidelines and included oral contraceptive pills, injectables, implants, intrauterine devices (IUDs), and condoms. Independent variables included respondents’ age, education level, employment status, region, place of residence, number of living children, wealth index (derived via principal component analysis), and exposure to family planning information through media.

All candidate independent variables were first assessed using bivariate logistic regression. Variables that did not demonstrate statistical significance at the bivariate level—including education level, place of residence, region, and wealth quintile—were excluded from the multivariable model, in accordance with standard model-building procedures that promote parsimony and prevent overfitting. Therefore, only variables meeting the inclusion criteria were entered into the multivariable logistic regression and reported in the final model. Religion was not included as a variable because all respondents in the dataset were Muslims, resulting in no variability for statistical comparison.

### Data analysis

The analysis proceeded in three stages. First, we summarized participant characteristics and contraceptive prevalence using descriptive statistics. Next, we performed bivariate logistic regression to assess unadjusted associations, reporting these as crude odds ratios (CORs) with 95% confidence intervals (CIs). Finally, a multivariable logistic regression model was constructed to identify independent predictors of modern contraceptive use, with results presented as adjusted odds ratios (AORs) and 95% CIs. All analyses were conducted using SPSS Statistics (Version 24).

## Ethical considerations

The SHDS 2020 received ethical approval from the Somali National Bureau of Statistics and associated institutional review boards. Informed consent was obtained from all participants before participation, and the dataset was anonymized prior to public release. This study involved secondary analysis of publicly available data and therefore posed no ethical risk, adhering to confidentiality and responsible data use.

## Results

### Socioeconomic factors

Analysis of the 2020 Somali Health and Demographic Survey revealed clear patterns in the sociodemographic makeup of the study population. Women from both younger and older reproductive age groups were represented, with a substantial proportion in the older group. Educational attainment varied widely, ranging from no formal schooling to higher education; however, contraceptive use remained consistently low across all educational levels. Similarly, although participants were distributed across all wealth quintiles, higher economic status did not correspond to greater contraceptive utilization (Table 1).

**Table 1:** Sociodemographic characteristics of women aged 15–49 in Somalia, 2020.

Variable	Category	*N* (%)
Age group (years)	≤25 years	1,146 (42.4)
	≥26 years	1,558 (57.6)
Residence type	Urban	2,300 (85.1)
	Rural	379 (14.0)
	Nomadic	25 (0.9)
Region	Awdal	167 (6.2)
	Woqooyi Galbeed	264 (9.8)
	Togdheer	182 (6.7)
	Sool	163 (6.0)
	Sanaag	182 (6.7)
	Bari	104 (3.8)
	Nugaal	175 (6.5)
	Mudug	182 (6.7)
	Galgaduud	123 (4.5)
	Hiraan	86 (3.2)
	Middle Shabelle	52 (1.9)
	Banadir	828 (30.6)
	Bay	33 (1.2)
	Bakool	37 (1.4)
	Gedo	39 (1.4)
	Lower Juba	87 (3.2)
Education level	No education	684 (25.3)
	Primary	711 (26.3)
	Secondary	868 (32.1)
	Higher	441 (16.3)
Wealth quintile	Lowest	17 (0.6)
	Second	40 (1.5)
	Middle	261 (9.7)
	Fourth	698 (25.8)
	Highest	1,688 (62.4)

### Regional distribution of participants

Most women in the sample resided in urban areas, with very small proportions living in rural or nomadic settings. However, living in an urban setting did not translate into higher contraceptive use, suggesting that physical proximity to facilities does not necessarily ensure access or uptake. Representation across regions also varied, with the largest share of respondents coming from Banadir and smaller proportions from Somaliland, Puntland, and the southern regions. Despite these regional differences, modern contraceptive use remained uniformly low across the country (Table 1).

### Reproductive characteristics and health education

Reproductive health behaviors, particularly with respect to the intention to delay or avoid pregnancy, reveal alarming trends. The majority of women had never attempted to prevent or delay pregnancy, indicating minimal prior engagement with contraception. Exposure to family planning information within health facilities was limited. While usage of mobile phones and the internet was common, traditional sources of health information—such as newspapers, radio, and television—reached relatively few women (Table 2).

**Table 2:** Reproductive health characteristics and awareness of women aged 15–49 in Somalia, 2020.

Variable	Category	*N* (%)
Ever tried anything to delay or avoid pregnancy	No	2,680 (99.1)
	Yes	24 (0.9)
Used the internet last month	No	144 (5.3)
	Yes	2,560 (94.7)
Told about family planning at a health facility	No	2,335 (86.4)
	Yes	369 (13.6)
Reading a newspaper or magazine	No	2,106 (77.9)
	Yes	598 (22.1)
Heard family planning on the radio in the last few months	No	2,375 (87.8)
	Yes	329 (12.2)
Heard family planning on TV in the last few months	No	2,315 (85.6)
	Yes	389 (14.4)
Heard family planning in newspaper/magazine in the last few months	No	2,531 (93.6)
	Yes	173 (6.4)
Heard family planning by text messages on mobile phone	No	943 (34.9)
	Yes	1,761 (65.1)
Heard family planning by text messages on social media	No	941 (34.8)
	Yes	1,763 (65.2)
Heard about family planning through text messages on CHW	No	852 (31.5)
	Yes	1,855 (68.5)

### Prevalence of modern contraceptive utilization and methods used

Modern contraceptive use was very rare among the study population, with only **1.8%** of women reporting current use. Among this small group of users, short-acting methods were the most commonly used, while long-acting options such as implants and intrauterine devices were used by only a few women. This pattern reflects both the limited availability of modern methods and the low level of awareness and access across the country (Table 3).

**Table 3:** Prevalence of modern contraceptive utilization and methods used.

Variable	Category	*N* (%)
Contraceptive use	Non-user	2,656 (98.2)
	Using modern method	48 (1.8)
Current contraceptive	Not currently using	2,656 (98.2)
	IUD	1 (0.04)
	Injectables	3 (0.11)
	Implants	6 (0.22)
	Pills	35 (1.3)
	Lactational amenorrhea method	3 (0.11)

### Multivariable regression analysis of factors associated with contraceptive use

The multivariable regression analysis identified significant associations between modern contraceptive use and several important factors, including age, health status, and media exposure. Women aged ≥26 years were 10 times more likely to use modern contraceptive methods compared to women with ≤25 years (OR: 10.132, 95% CI: 2.389–42.968, *p* = 0.002), suggesting that older women may have good awareness and greater access to family planning services compared to younger women. Moreover, women who had ever tried to delay pregnancy have higher odds of using contraceptive methods (OR: 0.106, 95% CI: 0.036–0.312, *p* < 0.001). Health education on the importance of modern contraceptive use was an essential factor. Women who heard about family planning at a health facility were twice as likely to use modern contraceptive methods compared to those who did not (AOR: 1.992, 95% CI: 1.023–3.877, *p* = 0.043). In addition, exposure to family planning education through media platforms such as radio (AOR: 0.442, 95% CI: 0.183–1.072, *p* = 0.071), television (AOR: 0.318, 95% CI: 0.147–0.688, *p* = **<**0.01), and newspaper or magazine (AOR: 2.145, 95% CI: 0.888–5.182, *p* = 0.090) was associated with higher odds of using modern contraceptive methods (Table 4).

**Table 4:** Multivariable regression analysis of factors associated with modern contraceptive use.

Variable	Contraceptive use, *N* (%)		COR (95% CI)	AOR (95% CI)	*P*-value
	User	Non-user			
Age					
≤25 years	2 (0.17)	1,144 (99.83)	1	1	<0.01
≥26 years	46 (2.95)	1,512 (97.05)	17.402 (4.216–71.836)	10.132 (2.389 42.968)	
Ever tried to delay or avoid pregnancy					
Yes	6 (25.0)	18 (75.0)	1	1	<0.01
No	42 (1.6)	2,638 (98.4)	0.048 (0.018– 0.126)	0.106 (0.036–0.312)	
At health facility, told of family planning					
Yes	21 (5.7)	348 (94.3)	1	1	0.045
No	27 (1.2)	2,308 (98.8)	0.194 (0.108–0.347)	1.992 (1.023–3.877)	
Heard family planning on radio last few months					
Yes	15 (4.6)	314 (95.4)	1	1	0.064
No	33 (1.4)	2,342 (98.6)	0.295 (0.158–0.549)	0.442 (0.183–1.072)	
Heard family planning on TV last few months					
Yes	24 (6.2)	365 (93.8)	1	1	<0.01
No	24 (1.0)	2,291 (99.0)	0.159 (0.090–0.284)	0.318 (0.147–0.688)	
Heard family planning in newspaper/magazine last few months					
Yes	1,742 (98.81)	21 (1.19)	0.173 (0.090–0.333)	2.145 (0.888–5.182)	0.09
No	914 (97.13)	27 (2.87)	1	1	

## Discussion

This study addresses a critical evidence gap by examining the determinants of modern contraceptive use in Somalia using nationally representative data from the 2020 SDHS. The analysis revealed an exceptionally low prevalence of modern contraceptive use at only 1.8%, placing Somalia far behind its regional counterparts—Sub-Saharan Africa (18.36%), Ethiopia (45.7%), and Kenya and Uganda (43.2%) (19–22). This pronounced disparity underscores the persistent and context-specific challenges facing Somalia despite global reproductive health initiatives. These challenges are driven by a complex interplay of strong sociocultural and religious norms, widespread misconceptions regarding contraceptive safety, and a fragile healthcare infrastructure that continues to limit access to family planning services (18, 23).

Somalia's health system faces significant structural barriers that directly limit access to family planning services. The 2022–2023 Somalia Harmonized Health Facility Assessment (HHFA) identified 1,215 operational health facilities, equivalent to one primary health facility per 17,200 people and one hospital per 98,000 people. Many of these facilities lack essential equipment, and service delivery relies heavily on UN agencies, international NGOs, and the private sector. These constraints reduce the availability, quality, and consistency of reproductive health services, thereby affecting women's access to accurate information and modern contraceptive methods (24).

Emerging evidence from SHDS 2020 has identified critical sociodemographic and regional disparities influencing contraceptive intention in Somalia (14, 17). For instance, the study showed higher intention to use contraception among educated and urban-dwelling women, particularly in Woqooyi Galbeed (18.4%), compared to 1.1% in the Gedo region, suggesting entrenched inequality in awareness and access. However, existing studies have focused mainly on intention rather than actual contraceptive use, leaving critical knowledge gaps regarding the determinants of modern contraceptive utilization (15, 25).

Age emerged as the strongest predictor of modern contraceptive use. Women aged 26 years and older had more than ten times the odds of using a modern method compared with younger women. This result is consistent with findings from Egypt (26), Uganda (22), and Pakistan (27), which suggests that with age, life experience, and prior childbirth, women may gain the knowledge, autonomy, and motivation to manage their fertility (5, 8, 27).

Exposure to family planning information through media—particularly television—also showed an important association with contraceptive use. In the adjusted model, women who heard family planning messages on television had significantly higher odds of using modern contraception. Although radio and newspaper exposure did not reach statistical significance after adjustment, their crude associations suggest that mass media remain an important, though currently underutilized, channel for disseminating reproductive health information in Somalia. This is consistent with evidence from many low- and middle-income countries, where media exposure has been shown to enhance contraceptive knowledge and uptake (28). Similarly, women who received family planning information at a health facility had almost twice the odds of using modern contraceptive methods. This suggests that direct, provider-led education remains a trusted source of accurate information, capable of addressing personal concerns and correcting misconceptions more effectively than general media messaging. This finding is consistent with evidence from Kenya, where women who discussed family planning with a health worker within the previous 12 months were 2.58 times more likely to use contraceptives compared with those who had not received such counseling (29).

The most compelling finding of this study is the paradox of what does not predict contraceptive use in Somalia. Contrary to extensive evidence across the region, including neighboring Kenya (27) and Ethiopia (10), socioeconomic advantages such as higher education, greater wealth, and urban residency were not significantly associated with contraceptive uptake (6, 8). However, in Tanzania, modern contraceptive use in rural areas increased by 44% because of sustained governmental efforts to promote family planning (30, 31). This suggests that in the Somali context, the protective effects of socioeconomic status are neutralized by more dominant, systemic barriers. These likely include pervasive sociocultural and religious norms that discourage contraception, limited male engagement, and the chronic disruption of health services, which undermines the reliable availability of methods and trained personnel, even in advantaged urban areas like Banadir. This reality explains the critical disconnect identified in our results: While facility-based education is highly effective, it fails to reach the vast majority of women, leaving a significant implementation gap (32).

The findings of this study should be interpreted with caution, considering several limitations. First, the cross-sectional design precludes establishing relationships between predictors and modern contraceptive use. Second, the dataset itself has inherent constraints: The exclusion of several regions due to security concerns may limit the generalizability of our results to the national level, while reliance on self-reported data introduces potential recall and social desirability bias. Finally, our analysis was limited to demand-side factors. It could not account for crucial supply-side variables, such as local contraceptive availability or healthcare provider attitudes, which are also critical determinants of uptake.

## Conclusion

This study confirms a critically low prevalence of modern contraceptive use among Somali women, with only 1.8% reporting current use. We identified older age and receiving information at a health facility as the most potent predictors of uptake, while, surprisingly, common indicators like urban residency, wealth, and education were not significant. These results highlight the urgent need to improve the quality and reliability of family planning services already available within primary healthcare, as this remains the most effective channel for influencing behavior. Moreover, broader health education through trusted community health workers and media is essential to build trust by directly countering pervasive misinformation, specifically addressing widespread fears about contraceptive safety and the critical misconception that it causes infertility, which continues to hinder progress, particularly among youth.

## Data Availability

The raw data supporting the conclusions of this article will be made available by the authors, without undue reservation.
